# A novel sweetpotato bZIP transcription factor gene, *IbbZIP1*, is involved in salt and drought tolerance in transgenic *Arabidopsis*

**DOI:** 10.1007/s00299-019-02441-x

**Published:** 2019-06-10

**Authors:** Chen Kang, Hong Zhai, Shaozhen He, Ning Zhao, Qingchang Liu

**Affiliations:** Key Laboratory of Sweetpotato Biology and Biotechnology, Ministry of Agriculture/Beijing Key Laboratory of Crop Genetic Improvement/Laboratory of Crop Heterosis and Utilization, Ministry of Education, College of Agronomy and Biotechnology, China Agricultural University, Beijing, 100193 China

**Keywords:** *Arabidopsis*, *IbbZIP1*, Salt and drought tolerance, Sweetpotato

## Abstract

**Key message:**

The overexpression of *IbbZIP1* leads to a significant upregulation of abiotic-related genes, suggesting that *IbbZIP1* gene confers salt and drought tolerance in transgenic *Arabidopsis*.

**Abstract:**

Basic region/leucine zipper motif (bZIP) transcription factors regulate flower development, seed maturation, pathogen defense, and stress signaling in plants. Here, we cloned a novel bZIP transcription factor gene, named *IbbZIP1*, from sweetpotato [*Ipomoea batatas* (L.) Lam.] line HVB-3. The full length of *IbbZIP1* exhibited transactivation activity in yeast. The expression of *IbbZIP1* in sweetpotato was strongly induced by NaCl, PEG6000, and abscisic acid (ABA). Its overexpression in *Arabidopsis* significantly enhanced salt and drought tolerance. Under salt and drought stresses, the transgenic *Arabidopsis* plants showed significant upregulation of the genes involved in ABA and proline biosynthesis and reactive oxygen species scavenging system, significant increase of ABA and proline contents and superoxide dismutase activity and significant decrease of H_2_O_2_ content. These results demonstrate that the *IbbZIP1* gene confers salt and drought tolerance in transgenic *Arabidopsis*. This study provides a novel bZIP gene for improving the tolerance of sweetpotato and other plants to abiotic stresses.

**Electronic supplementary material:**

The online version of this article (10.1007/s00299-019-02441-x) contains supplementary material, which is available to authorized users.

## Introduction

Salinity and drought seriously affect the productivity of agricultural crops in the world (Munns and Tester 2008; Yang et al. 2010; Zhao et al. 2013). Developing crops tolerant to salinity and drought is becoming important. Plants adapt to salinity and drought stresses by developing a variety of mechanisms, such as growth and development regulation, osmotic adjustment, detoxification, and ion homeostasis (Bohnert et al. 1995; Zhu 2001, 2002).

The basic region/leucine zipper motif (bZIP) proteins compose a large family of transcription factors in higher plants. The bZIP members have been reported to regulate flower development, seed maturation, pathogen defense and stress, light, hormone and sugar signaling pathways (Jakoby et al. 2002; Lindemose et al. 2013). Several *bZIP* genes have been found to confer the tolerance to abiotic stresses in some plant species. In *Arabidopsis*, *AtABF3*, *AtbZIP24,* and *AtbZIP1* are positive regulators of plant tolerance to abiotic stresses (Kim et al. 2004; Yang et al. 2009; Sun et al. 2012). In rice, *OsbZIP23*, *OsbZIP72*, *OsABF1*/*OsbZIP12*, *OsABF2*/*OsbZIP46,* and *OsbZIP71* increased the tolerance to abiotic stresses (Xiang et al. 2008; Lu et al. 2009; Hossain et al. 2010a, b; Liu et al. 2014a, b, c, d), but *OsbZIP52* negatively regulates responses to cold and drought (Liu et al. 2012). *GmbZIP44*, *GmbZIP62,* and *GmbZIP78* conferred salt and freezing tolerance in transgenic *Arabidopsis* (Liao et al. 2008). *GmbZIP1* increased the tolerance to salinity, cold temperature and drought in transgenic *Arabidopsis* and improved drought tolerance in transgenic wheat (Gao et al. 2011a, b). *ZmbZIP72* conferred salt and drought tolerance in transgenic *Arabidopsis* (Ying et al. 2012) and *ZmABP9* enhanced salt and drought tolerance in transgenic cotton (Wang et al. 2017).

Sweetpotato is an important food crop worldwide (Zang et al. 2009). Its productivity is often limited by salinity and drought stresses (Liu et al. 2014a). Gene engineering is an alternative approach for improving abiotic stresses’ tolerance in sweetpotato (Kim et al. 2012, 2013, 2014; Liu et al. 2014a, b, c, 2015; Wang et al. 2016a, b, c; Zhai et al. 2015; Li et al. 2017). To date, the bZIP family has not been reported in sweetpotato. Here, a novel bZIP transcription factor gene, named *IbbZIP1*, was isolated from sweetpotato and found to be involved in salt and drought tolerance in transgenic *Arabidopsis.*

## Materials and methods

### Plant materials

Sweetpotato line HVB-3 was used to isolate the *IbbZIP1* gene in this study. Its transcriptome sequencing was conducted by Li et al. (2017), from which one expressed sequence tag (EST) was obtained. *Arabidopsis thaliana* (Columbia-0, WT) was employed to analyze the function of *IbbZIP1*.

### Cloning and analysis of *IbbZIP1* and its promoter

Freshly-harvested storage roots of HVB-3 were used to extract total RNA. The first-strand cDNA was obtained using PrimeScript™ II 1st Strand cDNA Synthesis Kit (TaKaRa, Beijing, China). A rapid amplification of cDNA ends (RACE) procedure was applied to amplify the full-length cDNA of *IbbZIP1* with specific primers (Supplementary Table S1). Genomic DNA extracted from in vitro-grown plants was used to amplify the genomic sequence of *IbbZIP1* (Wang et al. 2016a). Its promoter was cloned with Universal GenomeWalker 2.0 Kit (TaKaRa, Dalian, China). The specific primers listed in Supplementary Table S1 were employed.

The *IbbZIP1* cDNA analysis was performed online (https://blast.ncbi.nlm.nih.gov/Blast.cgi). The ORF Finder (https://www.ncbi.nlm.nih.gov/orffinder/) was used to predict its open-reading frame (ORF). The phylogenetic analysis was conducted with the DNAMAN software (Lynnon Biosoft, Quebec, Canada). Exon–intron structure was constructed using Splign tool (https://www.ncbi.nlm.nih.gov/sutils/splign/splign.cgi?textpage=online&level=form) and compared with the *At1g58110* gene (http://www.arabidopsis.org/). The molecular weight and theoretical isoelectric point (*p*I) of the IbbZIP1 protein were analyzed online (http://web.expasy.org/compute_pi/). PlantCARE (http://bioinformatics.psb.ugent.be/webtools/plantcare/html/) was employed to identify the *cis*-acting regulatory elements in the promoter region of *IbbZIP1*.

### Expression analysis of *IbbZIP1* in sweetpotato

Storage root, stem, and leaf tissues of HVB-3 grown for 100 days in a field were used to isolate total RNA. The expression of *IbbZIP1* was analyzed by quantitative real-time PCR (qRT-PCR) with the specific primers of *IbbZIP1* (Supplementary Table S1) and *Ibactin* (AY905538) as an internal control based on the method of Liu et al. (2014a). Comparative *C*_T_ method was employed to quantify the gene expression (Schmittgen and Livak 2008). Furthermore, the HVB-3 plants cultured for 4 weeks on the basal Murashige and Skoog (MS) medium were transferred to liquid MS medium with H_2_O (control), 200 mM NaCl, 20% PEG6000 and 100 μM ABA, respectively, and sampled at 0, 2, 4, 6, 12, 24, and 48 h after treatment for analyzing the expression of *IbbZIP1*.

### Transactivation assay of IbbZIP1 in yeast

Transactivation assay of the IbbZIP1 protein in yeast (*Saccharomyces cerevisiae*) was done as described by Jiang et al. (2014). Its encoding region obtained with specific primers (Supplementary Table S1) was integrated into the vector pGBKT7 (pBD). Expression vector pBD-*IbbZIP1*, pGAL4 (as positive vector) and pBD (as negative vector) were transferred into the yeast strain AH109, respectively. The transactivation activity was determined according to the method of Wang et al. (2016a).

### Production of the transgenic *Arabidopsis* plants

The overexpression vector pC3301-121-*IbbZIP1* was constructed by inserting *35S*-*IbbZIP1*-*NOS* into pCAMBIA3301 at *Hind*III and *EcoR*I and then transferred into the *Agrobacterium tumefaciens* strain GV3101. The dipping flower method was applied to transform *Arabidopsis* (Clough and Bent 1998). The transgenic *Arabidopsis* plants were determined by histochemical GUS assay (Jefferson et al. 1987) and PCR analysis with 35S forward and *IbbZIP1*-specific reverse primers (Supplementary Table S1).

### Assay for salt and drought tolerance

Transgenic *Arabidopsis* T_3_ and WT seedlings were treated on MS medium containing 200 mM NaCl or 300 mM mannitol under 13 h day-light at 54 μM/m^2^/s and 22 °C. After 2 weeks, their root length and fresh weight were investigated. Furthermore, the T_3_ and WT seedlings were planted for 2 weeks in pots with a soil, vermiculite, and humus mixture (1:1:1, v/v/v) and each pot was irrigated with a 33 mL of 300 mM NaCl solution once every 2 days for 2 weeks, or stressed by drought for 4 weeks and re-watered for 2 days. The contents of abscisic acid (ABA) and proline and the activity of superoxide dismutase (SOD) in the T_3_ and WT plants treated in pots for 4 weeks under no stresses, 1 week under 300 mM NaCl stress or 2 weeks under drought stress were determined as described by Gao et al. (2011a). The H_2_O_2_ content was measured with H_2_O_2_ Assay Kit (Comin Biotechnology Co., Ltd. Suzhou, China). Twenty-seven plants in three pots with nine plants per pot were treated for each line.

### Assay for ABA sensitivity

For ABA sensitivity assay, the transgenic *Arabidopsis* and WT seeds (50 seeds for each line) were sown on MS medium with 0, 0.5, and 1 μM ABA, respectively, under 13 h day-light at 54 μM/m^2^/s and 22 °C. After 1 week, their germination rate and cotyledon opening and greening rate were investigated.

### Expression analysis of the related genes

The T_3_ and WT plants potted for 4 weeks without stress, 1 week stressed with 300 mM NaCl or 2 weeks stressed by drought were employed to analyze the expression of the genes involved in ABA and proline biosynthesis and reactive oxygen species (ROS) scavenging system using qRT-PCR protocols of Liu et al. (2014a). The specific primers of *Atactin* (internal control, NM112764) and the related genes were listed in Supplementary Table S1.

### Statistical analysis

All experiments were performed with three biological replicates. Data presented as the mean ± SE were analyzed by Student’s *t* test (two-tailed analysis) at *P* < 0.05 (*) and *P* < 0.01 (**).

## Results

### Cloning and sequence analysis of *IbbZIP1* and its promoter

**Fig. 1:**
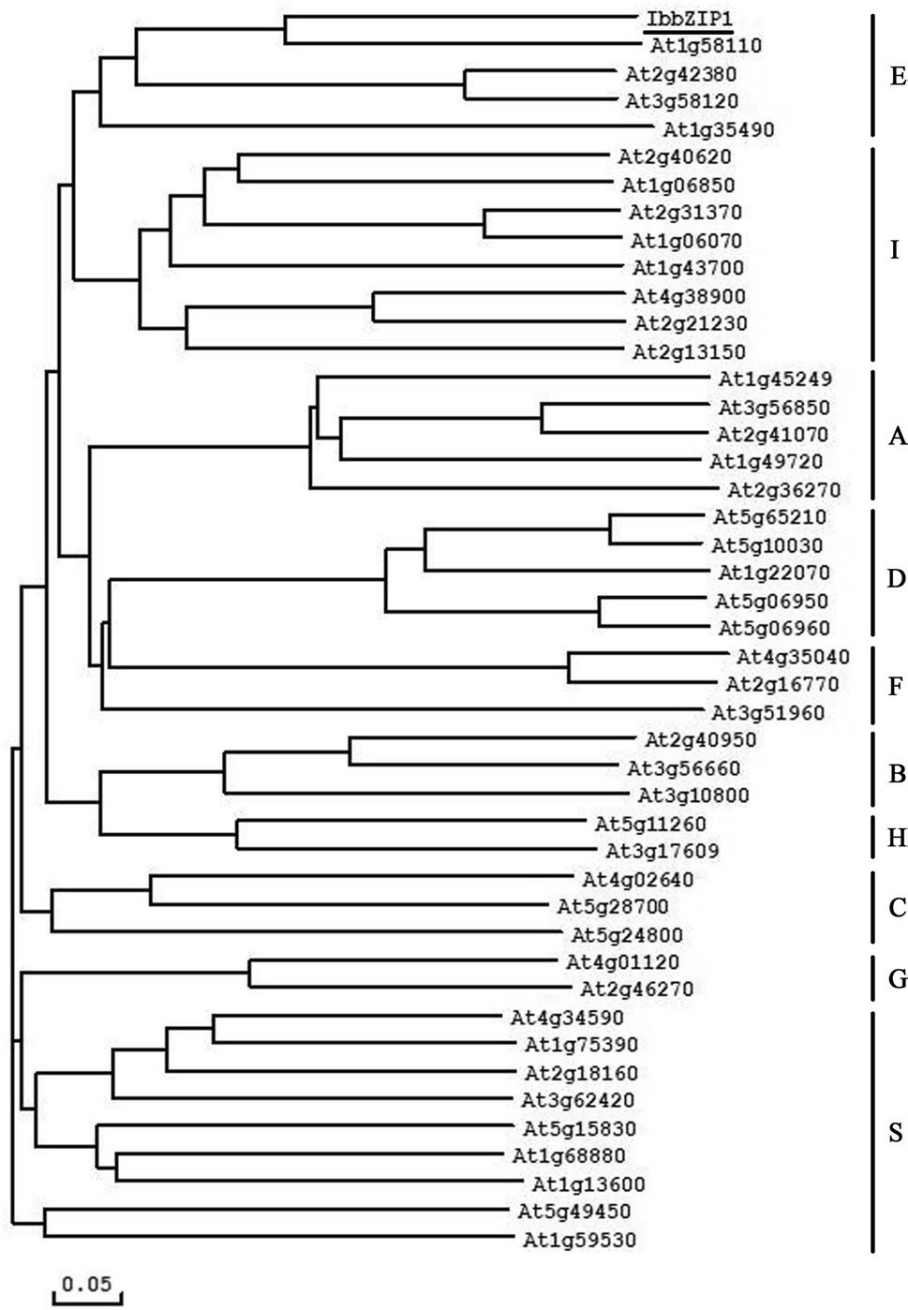
Phylogenetic analysis of IbbZIP1 with 44 *Arabidopsis* bZIP proteins. Ten groups of bZIP proteins were defined based on the classification of *Arabidopsis* bZIP proteins (Jakoby et al. 2002)

**Fig. 2:**
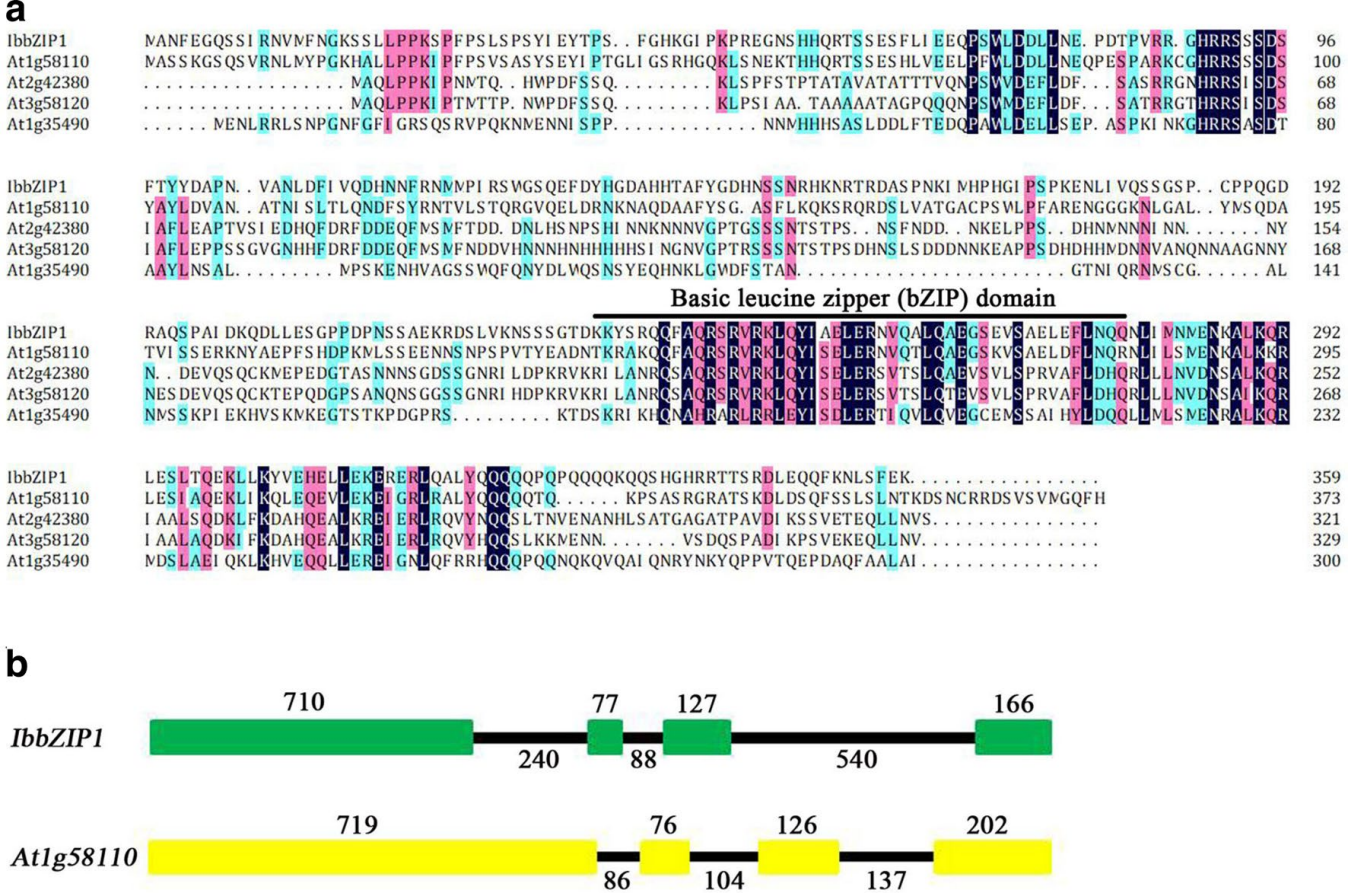
Comparison between IbbZIP1 and the *Arabidopsis* E group members. **a** Sequence alignment of IbbZIP1 with *Arabidopsis* proteins belonging to the E group. Black line shows the basic leucine zipper (bZIP) domain. **b** Comparison of exon and intron constituents between *IbbZIP1* and *At1g58110*. Exons are represented by colorful boxes and introns by black lines, with length (bp) displayed above exons and below introns

The *IbbZIP1* gene was cloned from sweetpotato line HVB-3. The cDNA sequence was 1757 bp in length and contained an 1080 bp ORF encoding a 359-aa polypeptide with a molecular weight of 41.26 kDa and a predicted *p*I of 7.89. The IbbZIP1 protein was subjected to phylogenetic analysis together with 44 *Arabidopsis* bZIP proteins belonging to 10 groups (Jakoby et al. 2002). The results revealed that IbbZIP1 belonged to group E of the bZIP family and had a close relationship with At1g58110 (Fig. 1). IbbZIP1 contained one bZIP domain (Fig. 2a). The 1946 bp genomic DNA of *IbbZIP1* contained 4 exons and 3 introns (Fig. 2b). Its promoter region (~ 2056 bp) had the stress-responsive *cis*-acting regulatory elements, such as HSE, MBS, TCA, GARE, TGA, ERE, and ABRE (Supplementary Fig. S1; Supplementary Table S2).

### Expression of *IbbZIP1* in sweetpotato

**Fig. 3:**
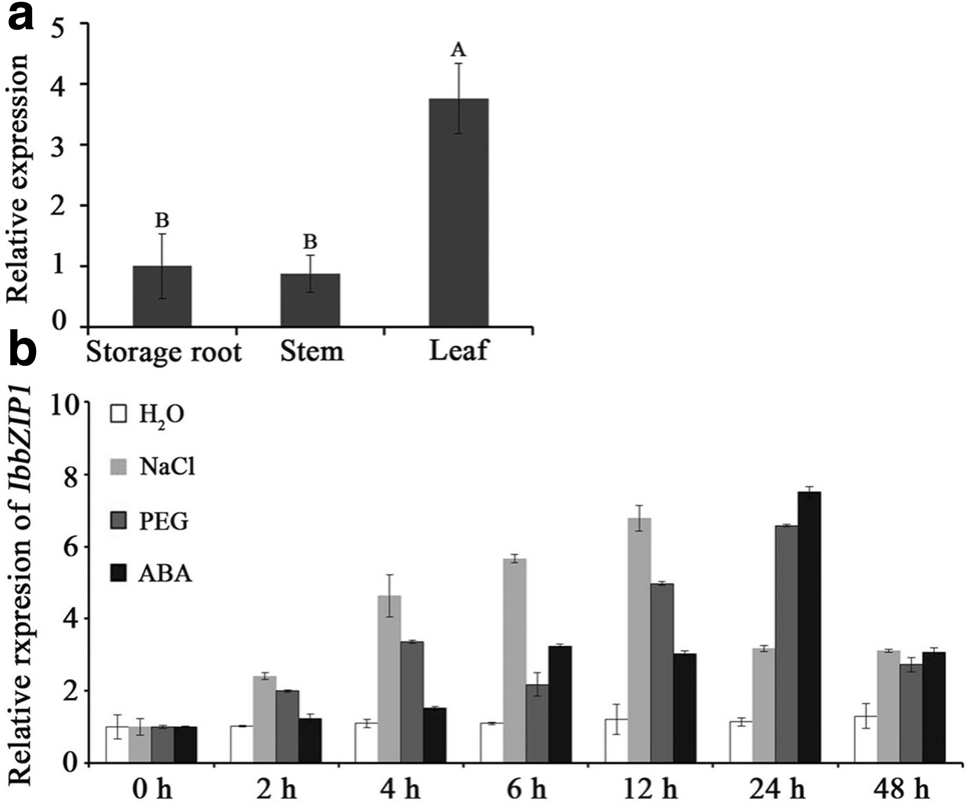
**a** Expression analysis of *IbbZIP1* in storage root, stem and leaf tissues of HVB-3. Different capital letters indicate a significant difference at *P *< 0.01 by Student’s *t* test. **b** Expression analysis of the *IbbZIP1* gene in the in vitro-grown plants of HVB-3 after different times (h) in response to H_2_O, 200 mM NaCl, 20% PEG6000 and 100 μM ABA, respectively. Data are presented as mean values ± SE (*n* = 3)

The *IbbZIP1* gene exhibited significantly higher expression level in the leaves of HVB-3 than in the stems and storage roots (Fig. 3a). Its expression in the in vitro-grown plants of HVB-3 was strongly induced by NaCl, PEG6000 and ABA, and peaked (6.79-fold) at 12 h of 200 mM NaCl treatment, (6.58-fold) at 24 h of 20% PEG6000 treatment and (7.51-fold) at 24 h of 100 μM ABA treatment (Fig. 3b). These results showed that *IbbZIP1* might be involved in the tolerance to salt and drought in sweetpotato.

### Transactivation activity of IbbZIP1 in yeast

**Fig. 4:**
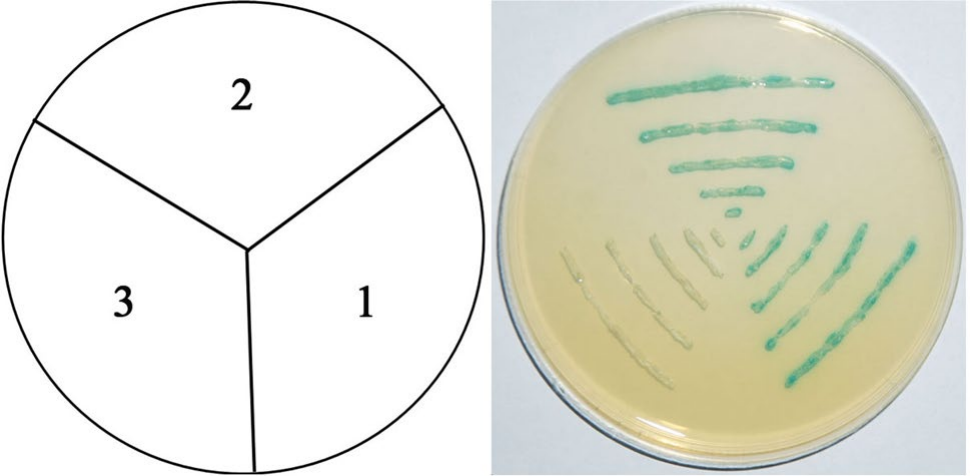
Transactivation assay of IbbZIP1 in yeast. (1) the pGAL4 vector as positive control. (2) pBD-IbbZIP1. (3) the empty pBD vector as negative control. The culture solution of the transformed yeast was drawn onto the SD plate without tryptophan and histidine

The yeast one-hybrid system was applied to identify a possible transactivation activity of the IbbZIP1 protein. The yeast cells harboring pGAL4 and pGBKT7-*IbbZIP1* grew well on SD plate without tryptophan and histidine and exhibited β-galactosidase activity, but the cells bearing pBD failed to grow (Fig. 4). These results demonstrated that IbbZIP1 had transactivation activity in yeast and has potential ability to activate the transcription of related genes by directly binding to *cis*-acting elements in their promoter region or interacting with other proteins.

### Production of the transgenic *Arabidopsis* plants

Lots of putatively transgenic *Arabidopsis* seeds were obtained by the dipping flower method. The randomly sampled seeds were sown on MS medium with 12.5 mg/L phosphinothricin (PPT) and produced plants. GUS assay and PCR analysis confirmed that 8 of the randomly sampled 60 plants were transgenic plants, named L1, L2, …, L8, respectively (Supplementary Fig. S2), from which T_3_ were generated. The expression of *IbbZIP1* in the leaves of the T_3_ and WT plants was analyzed by qRT-PCR and 4 of them (L1, L3, L6 and L8) exhibited significantly higher expression level compared with other plants (Supplementary Fig. S2).

### Enhanced salt and drought tolerance of transgenic *Arabidopsis*

**Fig. 5:**
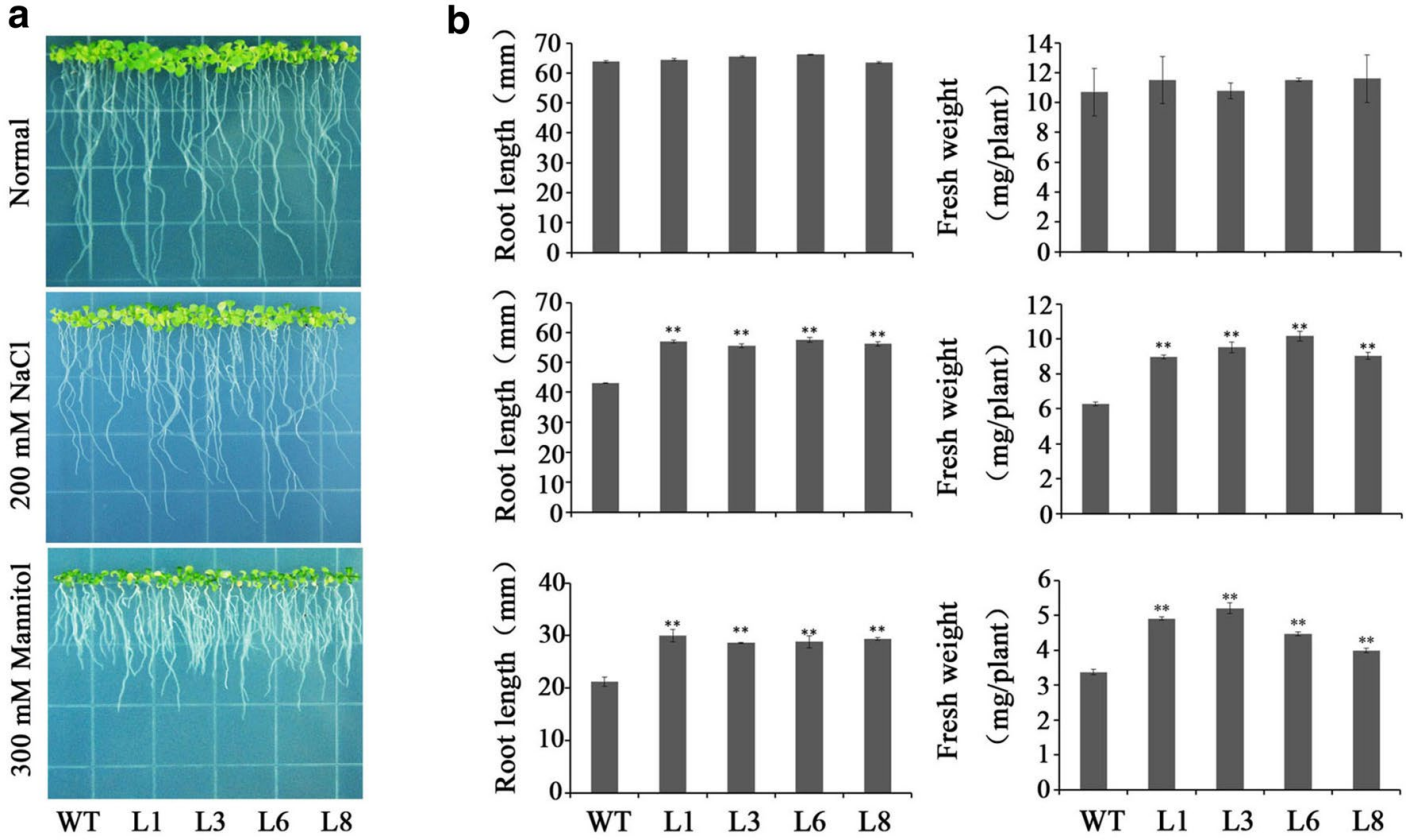
Responses of the transgenic *Arabidopsis* seedlings and WT cultured for 2 weeks on MS medium with 200 mM NaCl and 300 mM mannitol, respectively. **a** Growth and rooting of the transgenic *Arabidopsis* seedlings and WT. **b** Root length and plant fresh weight of the transgenic *Arabidopsis* seedlings and WT. Data are presented as mean ± SE (*n* = 3)

Four transgenic lines (L1, L3, L6, and L8) and WT seedlings were cultured on MS medium with 200 mM NaCl, 300 mM mannitol or without stresses for 2 weeks, respectively. The transgenic and WT plants exhibited no differences in growth without stresses, but the transgenic plants showed significantly better growth and increased physical size than WT under NaCl and mannitol stresses (Fig. 5).

**Fig. 6:**
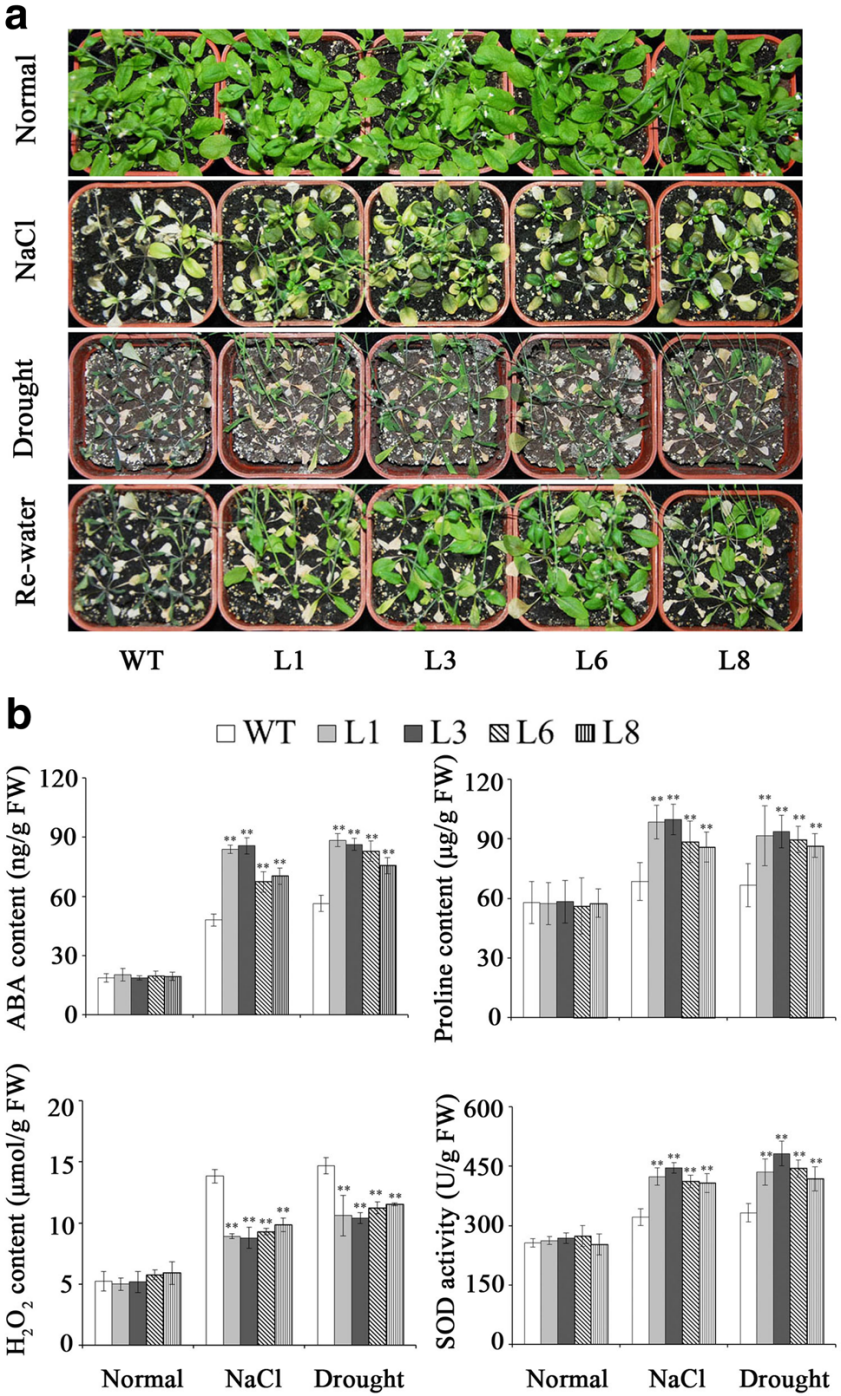
Responses of the transgenic *Arabidopsis* plants and WT grown in pots under salt and drought stresses. **a** Phenotypes of transgenic vs. WT plants grown for 6 weeks under normal condition, 2 weeks under 300 mM NaCl stress and 4 weeks under drought stress and then 2 days after re-watering, respectively. **b** ABA, proline, and H_2_O_2_ contents and SOD activity in the transgenic plants and WT grown for 4 weeks under normal condition, 1 weeks under 300 mM NaCl and 2 weeks under drought stress. Data are presented as mean ± SE (*n* = 3)

Two-week-old transgenic and WT plants grown in pots were stressed by 300 mM NaCl and drought, respectively. The transgenic plants and WT showed no differences in growth without stresses (Fig. 6a). Under salt and drought stresses, the transgenic plants exhibited good growth, increased ABA and proline contents, enhanced SOD activity and decreased H_2_O_2_ content, while WT almost died (Fig. 6a, b). These results indicated that L1, L3, L6, and L8 had significantly enhanced salt and drought tolerance compared with WT.

### Increased ABA sensitivity of transgenic *Arabidopsis*

**Fig. 7:**
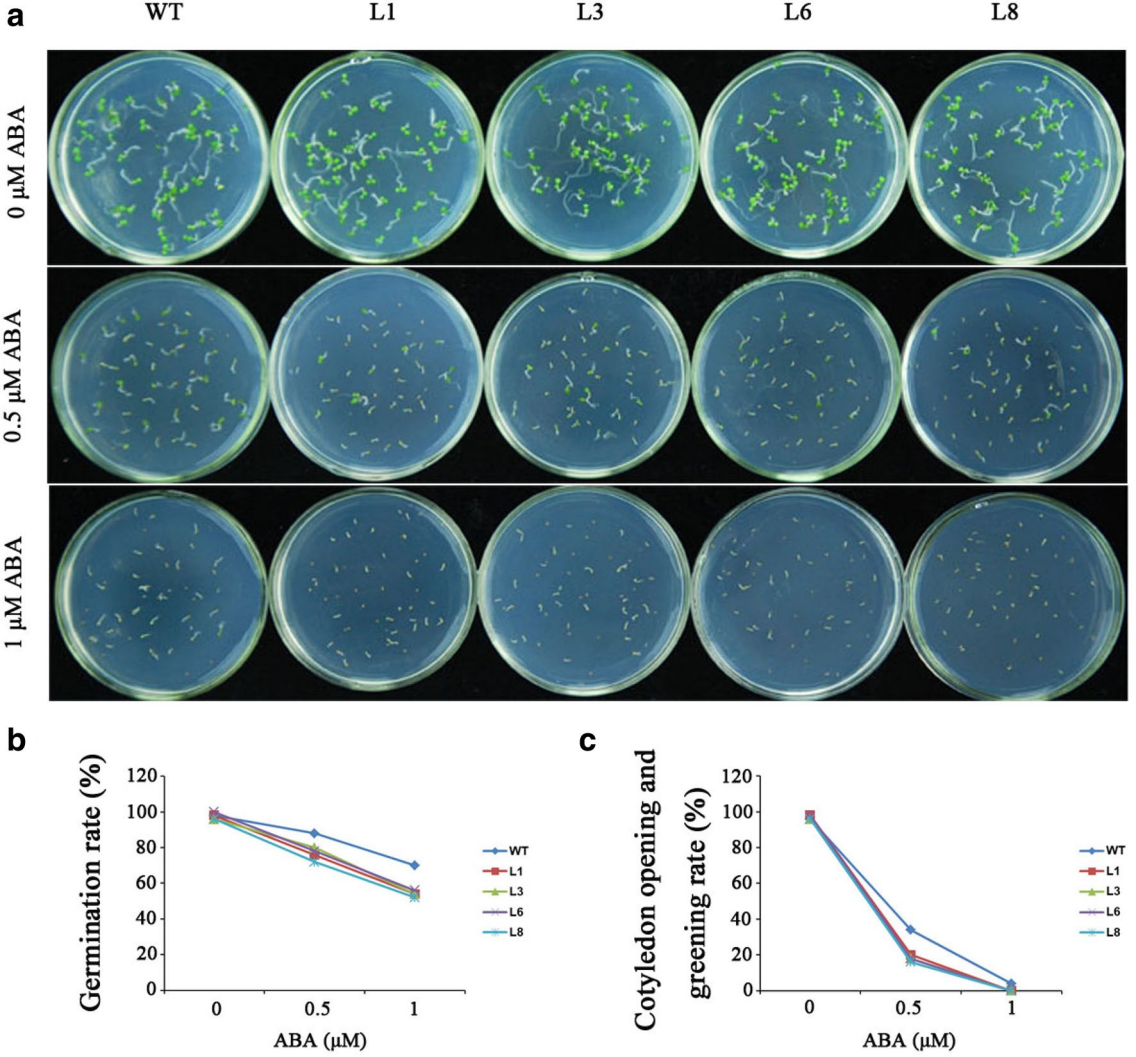
Responses of the transgenic *Arabidopsis* seeds and WT sown on MS medium with 0, 0.5, and 1 μM ABA for 1 week. **a** Growth vigor of the transgenic and WT seedlings. **b** Germination rates of the transgenic *Arabidopsis* seeds and WT. **c** Cotyledon opening and greening rates of the transgenic *Arabidopsis* seeds and WT

Seed germination in response to ABA was tested to determine whether *IbbZIP1* is involved in the ABA signaling pathway. No obvious differences in germination rate and cotyledon opening and greening rate were observed between the transgenic lines (L1, L3, L6, and L8) and WT under normal condition (Fig. 7). With exposure to different concentrations of ABA, both germination rate and cotyledon opening and greening rate of the transgenic and WT seeds declined, but the germination of L1, L3, L6, and L8 seeds were more sensitive to ABA-elicited inhibition, indicating that this gene might be involved in the ABA signaling pathway (Fig. 7).

### Expression of the stress-responsive genes in the transgenic *Arabidopsis* plants

**Fig. 8:**
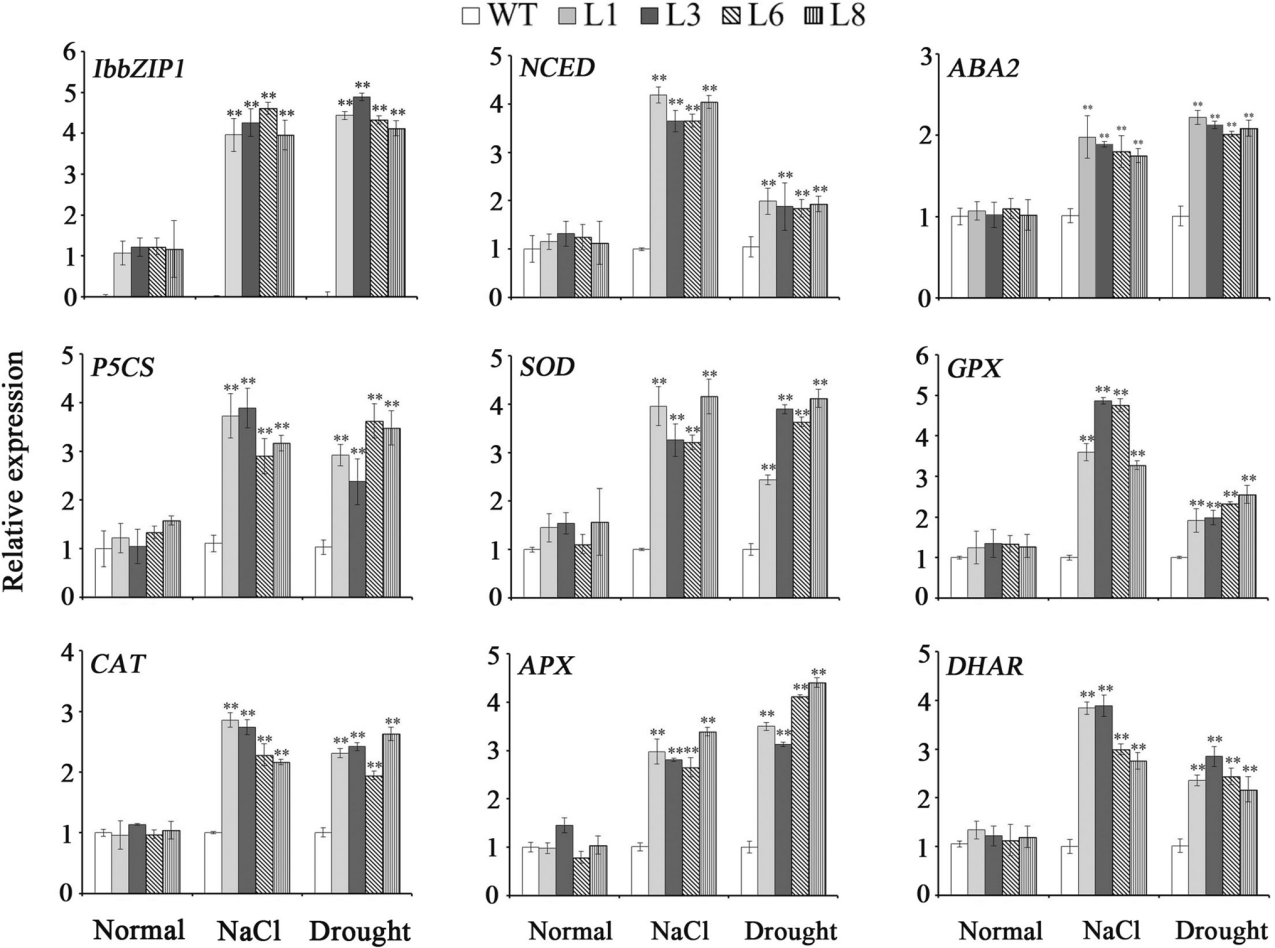
Transcript levels of salt- and drought-responsive genes in the transgenic *Arabidopsis* plants and WT under salt and drought stresses. Leaves of transgenic and WT pot-grown plants incubated for 4 weeks under normal condition, 2 weeks under 300 mM NaCl and 4 weeks under drought stress were used for expression analysis of the genes. Data are presented as mean ± SE (*n* = 3)

The genes encoding ABA biosynthetic 9-*cis*-epoxycarotenoid dioxygenase (NCED) and xanthoxin dehydrogenase (ABA2) and proline biosynthetic pyrroline-5-carboxylate synthase (P5CS) showed significantly higher expression levels in the transgenic plants in comparison with WT under salt and drought stresses (Fig. 8). The ROS scavenging genes encoding SOD, glutathione peroxidase (GPX), catalase (CAT), ascorbate peroxidase (APX) and dehydroascorbate reductase (DHAR) were found to show a systematic upregulation in the transgenic plants under salt and drought stresses (Fig. 8).

## Discussion

Several bZIP transcription factor genes have been cloned from *Arabidopsis*, rice, soybean, maize etc., and found to play roles in plant stress-responsive and hormone signal transduction (Sun et al. 2012; Liu et al. 2014a; Gao et al. 2011a; Wang et al. 2017). However, there is no report on the bZIP transcription factors in sweetpotato. This study reported, for the first time, the cloning and characterization of a novel transcription factor gene, *IbbZIP1*, from sweetpotato. The IbbZIP1 protein contains a typical bZIP domain which is essential for bZIP transcription factor, belongs to E group of bZIP family and has a close relationship with At1g58110 (Figs. 1, 2). To date, the function of the *At1g58110* gene is still unknown. We found that the promoter region of *IbbZIP1* had the stress-responsive *cis*-acting regulatory elements (Yamaguchi-Shinozaki et al. 2005, 2006; Xiang et al. 2008). The *IbbZIP1* expression was induced by NaCl, PEG, and ABA (Figs. S1, 4; Supplementary Table S2) and its overexpression increased salt and drought tolerance in transgenic *Arabidopsis* (Figs. 6, 7).

The bZIP transcription factors regulate the ABA-mediated abiotic stresses signaling pathways in plants (Mehrotra et al. 2014). In rice, overexpression of *OsABI5* and *OsbZIP23* upregulated the ABA biosynthetic gene *ABA2* (Zou et al. 2008; Xiang et al. 2008). The ABA biosynthetic gene *NCED2* was upregulated in the *ZmABP9*-overexpressing cotton plants (Wang et al. 2017). ABA regulates the expression of ABA dependent stress-responsive genes and higher levels of ABA can enhance salt and drought tolerance in *Arabidopsis* (Tuteja 2007; Liu et al. 2013; Wang et al. 2016a). In the present study, increased ABA sensitivity of transgenic *Arabidopsis* to exogenous ABA showed that *IbbZIP1* might be involved in the ABA signaling pathway (Fig. 7). The *IbbZIP1*-overexpressing *Arabidopsis* plants exhibited significant upregulation of *NCED* and *ABA2* and significant increase of ABA content under salt and drought stresses (Figs. 7, 8). It is suggested that overexpression of *IbbZIP1* confers tolerance to salt and drought due to the increased expression level of the ABA biosynthetic genes, which increases the production of ABA as a signaling molecule and further the expression of stress-responsive genes.

It has been reported that the high level of ABA in rice increases the transcript level of *OsP5CS1*, which lead to accumulation of proline under abiotic stresses (Sripinyowanich et al. 2013). It has been shown that proline accumulation resulted in the enhanced salt and drought tolerance in several plant species (Szabados and Savouré 2010; Krasensky and Jonak 2012; Zhang et al. 2012; Liu et al. 2015). Overexpression of *GmbZIP62* and *GmbZIP78* upregulated the genes involved in ABA signaling pathways and *GmP5CS1*, resulting the increased proline content and the enhanced salt and freezing tolerance in transgenic *Arabidopsis* (Liao et al. 2008). In this study, the *IbbZIP1*-overexpressing *Arabidopsis* plants exhibited the increased *P5CS* expression level and proline content under salt and drought stresses (Figs. 7, 8). These results suggested that more proline might be accumulated because of the increased ABA level, which leads to the enhanced salt and drought tolerance.

In plants, overproduction of ROS often occurs under salinity and drought stresses, which leads to oxidative damage. ROS can be detoxified by activating ROS scavenging enzymes (Gill and Tuteja 2010). The increased proline level led to upregulation of the ROS scavenging genes, which resulted in the enhanced tolerance to salt and drought in transgenic sweetpotato (Liu et al. 2014b, Liu et al. 2015; Wang et al. 2016b; Zhai et al. 2015). We found that under salt and drought stresses, the ROS scavenging genes (*SOD*, *GPX*, *CAT*, *APX,* and *DHAR*) were systematically upregulated in the *IbbZIP1*-overexpressing *Arabidopsis* plants (Fig. 8). Thus, more accumulation of proline in the *IbbZIP1*-overexpressing *Arabidopsis* plants might upregulate the ROS scavenging genes and further stimulate the ROS scavenging system, which results in the enhanced salt and drought tolerance.

In conclusion, a novel sweetpotato bZIP transcription factor gene, *IbbZIP1*, has been successfully isolated. The *IbbZIP1* gene is involved in salt and drought tolerance in transgenic *Arabidopsis*. Its overexpression might upregulate the genes involved in ABA and proline biosynthesis and ROS scavenging system and increase the ABA and proline contents, which leads to enhanced salt and drought tolerance. This study provides a novel bZIP gene for improving the tolerance of sweetpotato and other plants to abiotic stresses.

### Author contribution statement

Conceived and designed the experiments: QCL CK. Performed the experiments: CK. Analyzed the data: CK. Contributed reagents/materials/analysis tools: QCL HZ SZH NZ. Wrote the paper: QCL CK.

## Electronic supplementary material

Below is the link to the electronic supplementary material.

Supplementary material 1 (DOC 261 kb)

Supplementary material 2 (DOC 74 kb)

## Abbreviations

bZIP: Basic region/leucine zipper motif
EST: Expressed sequence tag
RACE: Rapid amplification of cDNA ends
*p*I: Isoelectric point
ABA: Abscisic acid
SOD: Superoxide dismutase
ROS: Reactive oxygen species
PPT: Phosphinothricin
