# Supplementary material for: A novel sweetpotato bZIP transcription factor gene, IbbZIP1, is involved in salt and drought tolerance in transgenic Arabidopsis
Source: Plant Cell Rep. 2019 Jun 10;38(11):1373–82. doi: 10.1007/s00299-019-02441-x (PMC6797668; doi:10.1007/s00299-019-02441-x)
Supplement: Supplementary file 1 — Supplementary material 1 (DOC 261 kb) [file 299_2019_2441_MOESM1_ESM.doc]

**
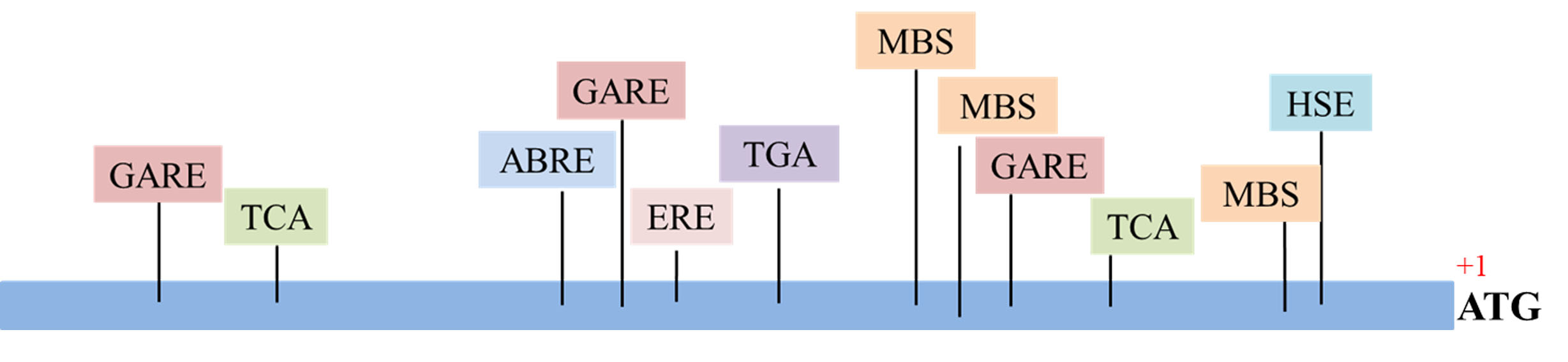
**

**Supplementary Fig. S1** The *IbbZIP1* promoter showing different *cis*-acting regulatory elements associated with abiotic stresses


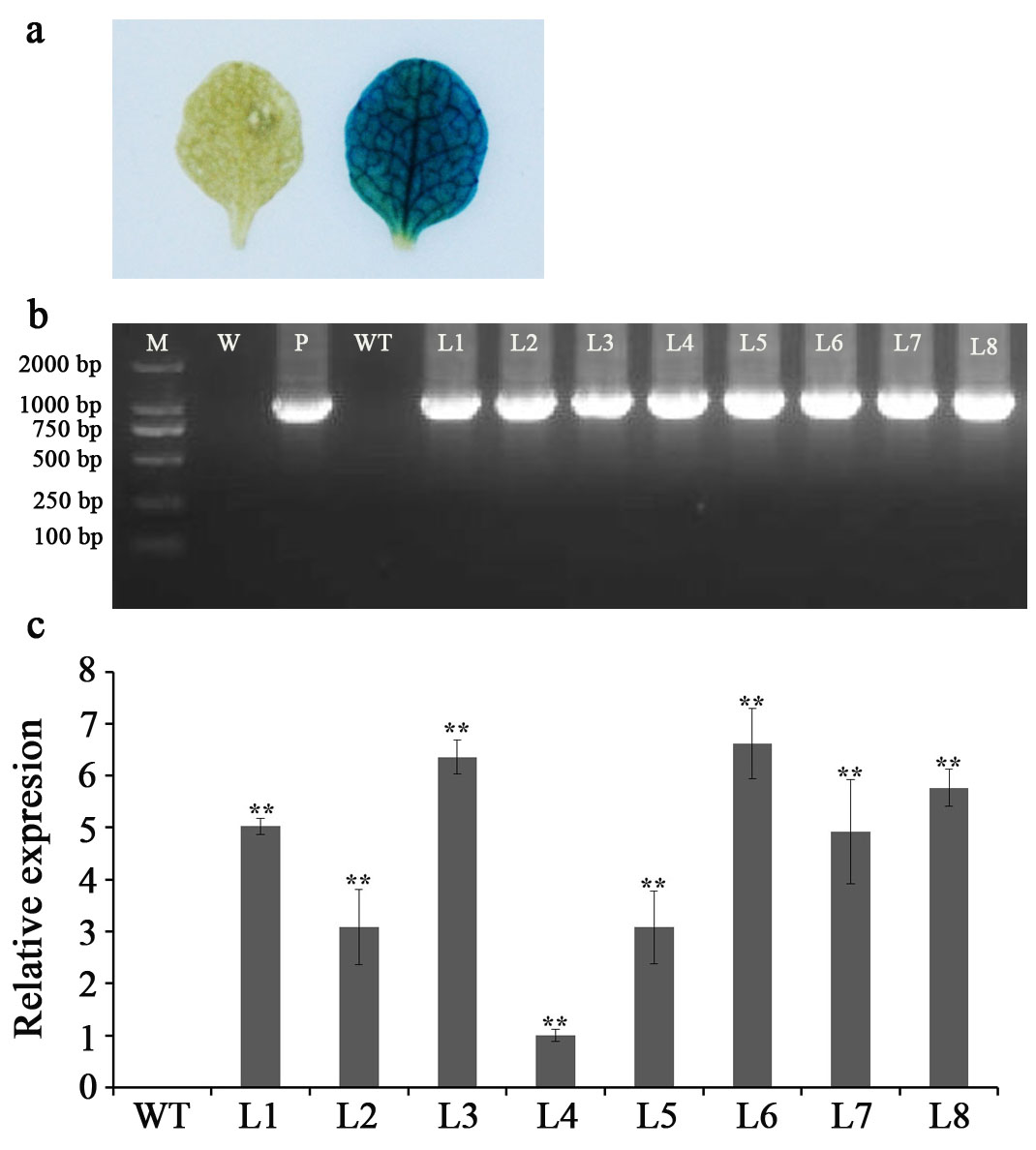


**Supplementary Fig. S2** Characterization of the *IbbZIP1*-overexpressing *Arabidopsis* plants. **a** GUS expression**. b** PCR analysis. M, DL2000 DNA marker; W, water as negative control; P, plasmid pCAMBIA3301-*IbbZIP1* as positive control; WT, negative control; L1-L8, transgenic plants. **c** Relative expression level of *IbbZIP1*. The *Arabidopsis actin* gene was used as an internal control. Data are presented as means ± SE (n=3)
