# Supplementary material for: A novel sweetpotato bZIP transcription factor gene, IbbZIP1, is involved in salt and drought tolerance in transgenic Arabidopsis
Source: Plant Cell Rep. 2019 Jun 10;38(11):1373–82. doi: 10.1007/s00299-019-02441-x (PMC6797668; doi:10.1007/s00299-019-02441-x)
Supplement: Supplementary file 2 — Supplementary material 2 (DOC 74 kb) [file 299_2019_2441_MOESM2_ESM.doc]

| **Supplementary Table S1** Primers used in this study | |
| --- | --- |
| Primer name | Primer sequence (5’-3’) |
| Primers for 5’/3’ RACE | |
| 5GSP1 | AGCGGAAACTTCAGAGCCT |
| 5GSP2 | ACATTCCTTTCCAGCTCAG |
| 5GSP3 | TTTTGGCATGAGGAGAATC |
| 3GSP1 | CTTCTTCTGGAACCGACAAAAAG |
| 3GSP2 | CATGAACATGGAGAATAAAGCGC |
| Primers for 5’-promoter region | |
| GW1 | TCCTTTTACATTCGTTGAAGTCCAT |
| GW2 | CTGGCTTGTATTCGTCGTCTTCT |
| Primers for constructing vectors | |
| OS-F-*Hind*III | CCCAAGCTTATGGCAAACTTTGAGGGACAATCTA |
| OS-R-*EcoR*I | GGAATTCTTATTTTTCAAAAGAGAGGTTTTTA |
| pBD-F-*Nde*I | GGAATTCCATATGATGGCAAACTTTGAGGGACAATCTA |
| pBD-R-*Sal*I | ACGCGTCGACTTATTTTTCAAAAGAGAGGTTTTTA |
| Primers for identifying transformants | |
| 35S-F | GAACTCGCCGTAAAGACTGG |
| *IbbZIP1*-R | TTATTTTTCAAAAGAGAGGTTTTTA |
| Primers for qRT-PCR | |
| *Ibactin*-F | AGCAGCATGAAGATTAAGGTTGTAGCAC |
| *Ibactin*-R | TGGAAAATTAGAAGCACTTCCTGTGAAC |
| *IbbZIP1*-F | ACCCCTTCTTTCGGTCACAA |
| *IbbZIP1*-R | GCCGATGACCTCTACGTACA |
| *Atactin*-F | GCACCCTGTTCTTCTTACCGA |
| *Atactin*-R | AGTAAGGTCACGTCCAGCAAGG |
| *AtNCED*-F | CGCCGGTTTAGTTTATTTCAATGGT |
| *AtNCED*-R | AATCGTACCGACCCGAAGTTTCTAA |
| *AtABA2*-F | TACTTGGGGTAAAGGGCGTG |
| *AtABA2*-R | CCAAGGACCCAGTCAAGCAT |
| *AtP5CS*-F | GCCTGATGCACTTGTTCAGA |
| *AtP5CS*-R | TTGAGCAATTCAGGGACCTC |
| *AtSOD*-F | ATGAGAAGTTCTATGAAGAG |
| *AtSOD*-R | GTCTTTATGTAATCTGGT |
| *AtGPX*-F | ATGGCGACGAAGGAACCAG |
| *AtGPX*-R | ATCGCCGAAGATTCCCCATTT |
| *AtCAT*-F | GCAACTACCCCGAGTGGAAA |
| *AtCAT*-R | TGTTCAGAACCAAGCGACCA |
| *AtAPX*-F | CTCTGGGACGATGCCACAAG |
| *AtAPX*-R | CTCGACCAAAGGACGGAAAA |
| *AtDHAR*-F | ATGGTCCTTTTATCGCCGGG |
| *AtDHAR*-R | GCCCATCCAGAGATCACACA |

**Supplementary Table S2** *Cis*-acting regulatory elements detected in the promoter region of the *IbbZIP1* gene

| Position | Name | Sequence | Predicted function |
| --- | --- | --- | --- |
| -1300^a^ | ABRE | CCTACGTGGC | Abscisic acid response |
| -1111 | ERE | ATTTCAAA | Ethylene response |
| -628 -1208 -1837 | GARE-motif | TCTGTTG | Gibberellin response |
| -806 -906 | TGACG-motif | CGTCA/TGACG | MeJA response |
| -520 -1636 | TCA-element | CCATCTTTTT | Salicylic acid response |
| -906 | TGA-box | TGACGTAA | Auxin response |
| -212 | HSE | AAAAAATTTC | Heat stress response |
| -722 -1636 | LTR | CCGAAA | Low temperature response |
| -318 -770 -804 | MBS | CAACTG | Drought stress response |
| -1071 | TC-rich repeats | ATTTTCTCCA | Defense and stress response |
| -1841 | Box-W1 | TTGACC | Fungal elicitor response |
| -850 | TA-rich region | TATATATATATA | Gene expression enhancer |
| -904 -1177 | O2-site | GTTGACGTGA | Zein metabolism regulation |
| -931 -1095 | Skn-1_motif | GTCAT | Endosperm expression element |

^a^ The first nucleotide “A” in the start codon was designated as +1
